# Preparation and Characterisation of Acid–Base-Change-Sensitive Binary Biopolymer Films with Olive Oil and Ozonated Olive Oil Nano/Microcapsules and Added Hibiscus Extract

**DOI:** 10.3390/ijms241411502

**Published:** 2023-07-15

**Authors:** Magdalena Janik, Karen Khachatryan, Gohar Khachatryan, Magdalena Krystyjan, Sandra Żarska, Wojciech Ciesielski

**Affiliations:** 1 Laboratory of Nanomaterials and Nanotechnology, Faculty of Food Technology, University of Agriculture, Balicka Street 122, 30-149 Krakow, Poland; magdalena.janik@student.urk.edu.pl; 2 Department of Food Quality Analysis and Assessment, Faculty of Food Technology, University of Agriculture, Balicka Street 122, 30-149 Krakow, Poland; gohar.khachatryan@urk.edu.pl; 3 Department of Carbohydrates Technology and Cereal Processing, Faculty of Food Technology, University of Agriculture, Balicka Street 122, 30-149 Krakow, Poland; magdalena.krystyjan@urk.edu.pl; 4 Faculty of Mathematics and Natural Sciences, Jan Dlugosz University in Czestochowa, 13/15 Armii Krajowej Ave., 42-200 Czestochowa, Poland; szarska007@gmail.com (S.Ż.); wc@ujd.edu.pl (W.C.)

**Keywords:** hibiscus, nano/microcapsules, alginate, chitosan

## Abstract

The purpose of this study was to develop and characterise bionanocomposites based on chitosan (CHIT) and alginate (ALG) in two series, which were subsequently functionalised with emulsions based on a combination of water, oil, ozonated oil and hibiscus flower extracts. The structure and morphology of the materials produced were characterised by Fourier transform infrared spectroscopy (FTIR), scanning electron microscopy (SEM) and ultraviolet and visible light (UV-Vis) absorption spectroscopy, along with a surface colour analysis and the determination of the mechanical and thermal properties of the resulting composites. Functionalisation did affect the analysed composite parameters. The FTIR spectra indicated that the polysaccharide matrix components were compatible. The SEM images also confirmed the presence of nano/microcapsules in the polysaccharide matrix. The obtained results indicate that the order of adding polysaccharides has a significant impact on the encapsulation capacity. The encapsulation resulted in the improved thermal stability of the composites. The emissions analysis showed that the composites containing nano/microcapsules are characterised by a higher emission intensity and are sensitive to acid or base changes. Significant differences in emission intensity were observed even at low concentrations of acids and bases. A drop in the mechanical properties was observed following functionalisation. The results of this study suggest that these bionanocomposites can be used as active and/or smart packaging materials.

## 1. Introduction

One of the methods for ensuring food safety is to use preservatives to extend the food life and achieve longer expiry dates. At present, food producers have a wide range of preservatives at their disposal, which are classified as food additives. The doses of such preservatives used to preserve food products are regulated by law. Furthermore, growing numbers of consumers are now seeking food without added preservatives, relying on their natural equivalents and, at the same time, still expecting them to be safe. The use of preservatives, which are generally synthetic substances [1], is a sound practice to ensure food safety. However, the use of natural preservatives, such as essential oils and plant extracts, offers a valid alternative. Such substances can be included in innovative packages based on natural polysaccharides as active and/or smart packages [2]. This type of packaging can have a beneficial effect on the product being stored. With its inclusion in packages of bioactive substances, such as those having an antioxidating or antimicrobial effect, food can gain a longer shelf life through a reduction in oxidating processes or the inhibition of microorganism growth [3]. The combination of the properties of natural compounds and the achievements of nanotechnology allows us to obtain materials with new, unique properties that can compete with plastics. Studying the properties of these materials is necessary to make the public aware of the advantages of using modern packaging materials [4,5].

The flowers, leaves and fruits of many plants have been used for consumption, medicine, therapy and decoration throughout all of human history. The bioactive properties of plants are linked to the presence of various groups of chemical compounds, including phenols, alkaloids, terpenoids, vitamins and pigments [6]. In recent years, we observed a rise in the number of studies concerning the use of plants and their derivative products as substitutes for or superior alternatives to chemical preservatives [7]. 

*Hibiscus sabdariffa* L. (family Malvaceae) is primarily grown in Central and West Africa, as well as in South-East Asia [8]. The primary group of chemical compounds that make up this plant are organic compounds and phenolic acids, including citric acid, malic acid, tartaric acid, oxalic acid and hibiscus acid, which is distinctive of this plant [9]. Additionally, hibiscus flowers are rich in anthocyans, polyphenols and flavonoids. Anthocyans confer hibiscus flowers with their distinctive, intense colour. For this reason, hibiscus extract is used to dye many food products [10]. Polyphenols and flavonoids are considered antioxidants that can protect the human body from infections. Hibiscus also forms a natural source of vitamin C [11]. Due to its properties, the vitamin has found a range of uses in many areas of everyday life, including the pharmaceutical and food industries. The most widely used part of this plant is the calyx [12]. The fresh or dried flowers of this plant are used to prepare hot or cold herbal drinks, fermented beverages, wines and sweet products, including jams, condiment jellies, ice cream, flavouring substances and cakes [13]. Extracts made of dried hibiscus petals show good antioxidating effects, lower elevated blood pressure and reduce the risk of diseases related to obesity, diabetes and the circulatory system [14,15]. It is also notable that *H. sabdariffa* extracts are characterised by a low acute toxicity (LD50 from 2000 to over 5000 mg/kg/day) [16]. The hibiscus flower extract has been used in nanotechnology as a reducer for producing nanoparticles synthesised using green chemistry methods, e.g., AuNPs [17], AgNPs [18,19] and CdO [20]. Due to such positive effects of the hibiscus flower extract, it constitutes a highly interesting and promising natural component for use in biopolymer films as an alternative to its synthetic equivalents. The hibiscus extract also shows interesting optical properties. Studies on the extract have shown that it is characterised by pronounced luminescent properties in both its natural and reduced forms. The extracted solution changes colour from bright purple to yellow following chemical reduction, with the emission wavelength changing as well [21].

Chitosan and alginate are two polysaccharides that, due to their many biological properties (antimicrobial, antioxidant, immunostimulating, anticarcinogenic, anti-inflammatory, biodegradable, non-toxic and biocompatible), have been the object of numerous studies in multiple fields of research, including food, biomedicine, pharmacy and cosmetology [22,23]. Alginates have been used to produce biodegradable films, but due to their poor mechanical properties and high sensitivity to water, their applications are severely limited [24]. For this reason, researchers continue to seek new solutions to improve the properties of biopolymer films using various modifications, including functional additions, such as plant extracts, essential oils and nanostructures [25]. According to the literature data, a complex of chitosan and alginate polyelectrolytes reduces the release of encapsulated substances and is less soluble in water than chitosan or alginate alone [26]. Thus, a combination of these two polysaccharides may be a useful solution from the perspective of designing and producing biopolymer films, which can be additionally enriched with active substances. 

Olive oil is a natural vegetable oil obtained from mechanically pressed olive tree fruit [27], something that has been known about since antiquity [28]. Due to its health properties, it is highly valued and regarded as a superfood [29]. Olive oil also shows various antibacterial properties. Scientists like Eduardo Medina, Antonio de Castro and others have studied the antimicrobial activity of various edible vegetable oils. Their results have demonstrated that olive oil has a strong bactericidal effect against a wide spectrum of microorganisms. Moreover, they report that the effect is stronger against Gram-positive bacteria than against Gram-negative ones [30]. There are many studies that have used olive oil to produce microcapsules [31]. Due to its natural origin and biological properties, it was used in this study to generate nano/microcapsules. Additionally, to reinforce the antimicrobial effect, the olive oil in this study was ozonated. According to the literature data, ozonated olive oil shows an antibacterial effect against Gram-positive bacteria, e.g., *Bacillus subtilis* and *Staphylococcus aureus*, and against Gram-negative ones, e.g., *Escherichia coli* and *Pseudomonas aeruginosa* [32]. In our previous paper, we noted that olive oil ozonation enables the use of the antibacterial potential of both these components [33]. 

Nowadays, scientists from various branches of industry seek modern systems for delivering bioactive compounds in a non-invasive and controlled manner. They achieve this by using microencapsulation techniques as a technology intended to improve the delivery strategy, controlled release and bioactive compound protection [34]. The encapsulation process can use various matrix types based on different polymers, including chitosan, which has an antibacterial effect and prevents food product spoilage. However, pure chitosan has poor mechanical and barrier properties, and it loses its antibacterial activity in environments with a pH above 6.5. This can be prevented by combining chitosan with other polymers [35]. To overcome the limitations of hydrophilic hydrocolloids, such as chitosan or alginate, they are often combined with lipids to produce multi-component or emulsion-based films. Many studies describe the incorporation of lipids (including plant and animal fats) into film-forming solutions. Vegetable oils are readily available, inexpensive, non-toxic, non-depletable and non-volatile [36]. The enclosure of bioactive components in micro/nanocapsules protects them against degradation due to external agents, such as light or permeation by gases during storage. Encapsulation can also stop unpleasant flavours and odours, further broadening the applicability in food technology and pharmaceuticals [37]. Coatings based on chitosan, sodium alginate and olive oil have been used as coatings for the cold storage of fresh figs. Studies have shown that the coatings were effective as a sub-technology for preserving the organoleptic and sensory qualities and bioactive components of figs during storage at low temperatures [38].

The purpose of this study was to produce and characterise the physicochemical and functional properties, with the microstructure of composite binary biopolymer films based on chitosan and sodium alginate containing nano/microcapsules of ozonated olive oil and hibiscus flower extract. An additional purpose was to investigate the effects of acidic and basic environments on the optical properties of the films.

## 2. Results and Discussion

### 2.1. FTIR-ATR Spectrophotometry of the Composites Obtained

**Figure 1:**
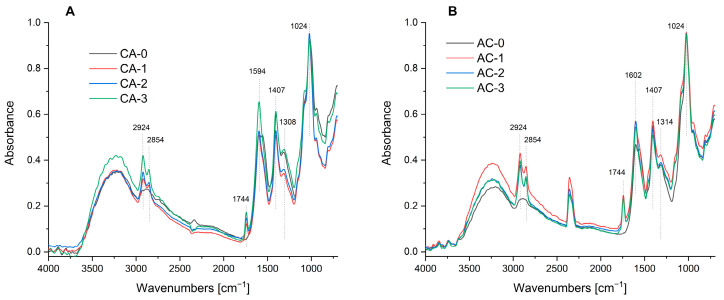
FTIR-ATR spectra of the obtained composites for CA (**A**) and AC (**B**) series.

FTIR spectroscopy made it possible to characterise the chemical structure of the composites obtained. The obtained spectra shown in Figure 1A,B are typical of chitosan and sodium alginate. The broad absorption band observed in the 3000–3500 cm^−1^ range is caused by the presence of hydroxyl (-OH) and amine (-NH_2_) groups. The distinctive chitosan/alginate complex band at a wave number of approx. 1600 cm^−1^ (1594 cm^−1^ for the CA series and 1602 cm^−1^ for the AC series) and the intense peaks located at approx. 1024 cm^−1^ indicate the presence of symmetric deformation vibrations of NH in the -NH_3_^+^ ion. These point to the forming of ionic bonds between the negatively charged carboxyl groups of sodium alginate and the positively charged amine groups of chitosan [39]. The presence of nanocapsules based on both regular and ozonated oil with hibiscus flower extract did not cause significant structural changes in the binary polysaccharide matrix. The bands at wave numbers 2924 cm^−1^ and 2854 cm^−1^ point to the presence of -CH_2_ and CH_3_ groups (asymmetrical and symmetrical stretching vibrations) from olive oil [40]. An increase in absorbance was observed in the AC series between 2923 cm^−1^ and 3245 cm^−1^, for which -OH groups are responsible. The band at wavelength 1744 cm^−1^ corresponds to the carbonyl functional group of triglycerides, which come from the olive oil present in the composites [41]. The higher intensity observed at the 1744 cm^−1^ peak indicates the higher accessibility of olive oil particles. It can be concluded on this basis that the stronger signal observed for the AC series is due to the fact that an oil leak on the composite surface occurred in this series, which was confirmed by the visual assessment of the test materials and by scanning electron microscopy. 

### 2.2. UV-Vis Spectroscopy

**Figure 2:**
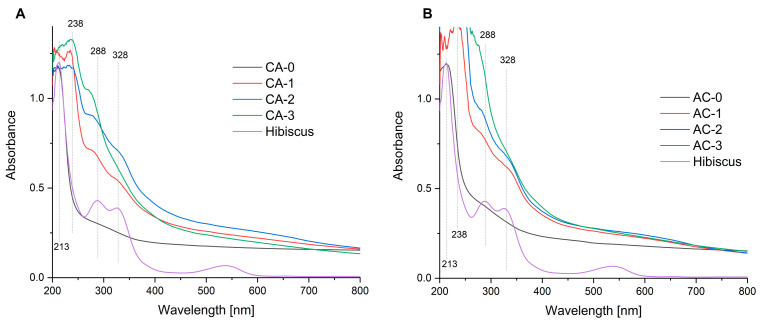
UV-Vis spectra of CA (**A**) and AC (**B**) series films, as well as the hibiscus extract spectrum.

Figure 2A,B show the absorption spectra of the films and of the hibiscus flower aqueous extract in the ultraviolet and visual range. The hibiscus extract is clear with a purple colour, which is visible in the absorption spectrum with a slightly higher absorption of the green wavelength (550 nm). Due to this absorption, the solution appears bright purple/red (50). The control composites did not exhibit radiation absorbance within the visual range, while the functionalisation performed in both series led to an increase in absorbance. The CA-3 film showed the highest absorbance in this group. The changes in the absorption spectra can be attributed to the binary polysaccharide complex functionalisation with the emulsions. Encapsulation causes an increase in absorbance within the 230 to 280 nm range [42]. The resulting band comes from the nano/microcapsules formed in the polysaccharide matrix. The increase in and deformation of the bands can be attributed to the effects of compounds originating from the hibiscus extract, caused by their interactions with the polysaccharide matrix.

### 2.3. Emissions

**Figure 3:**
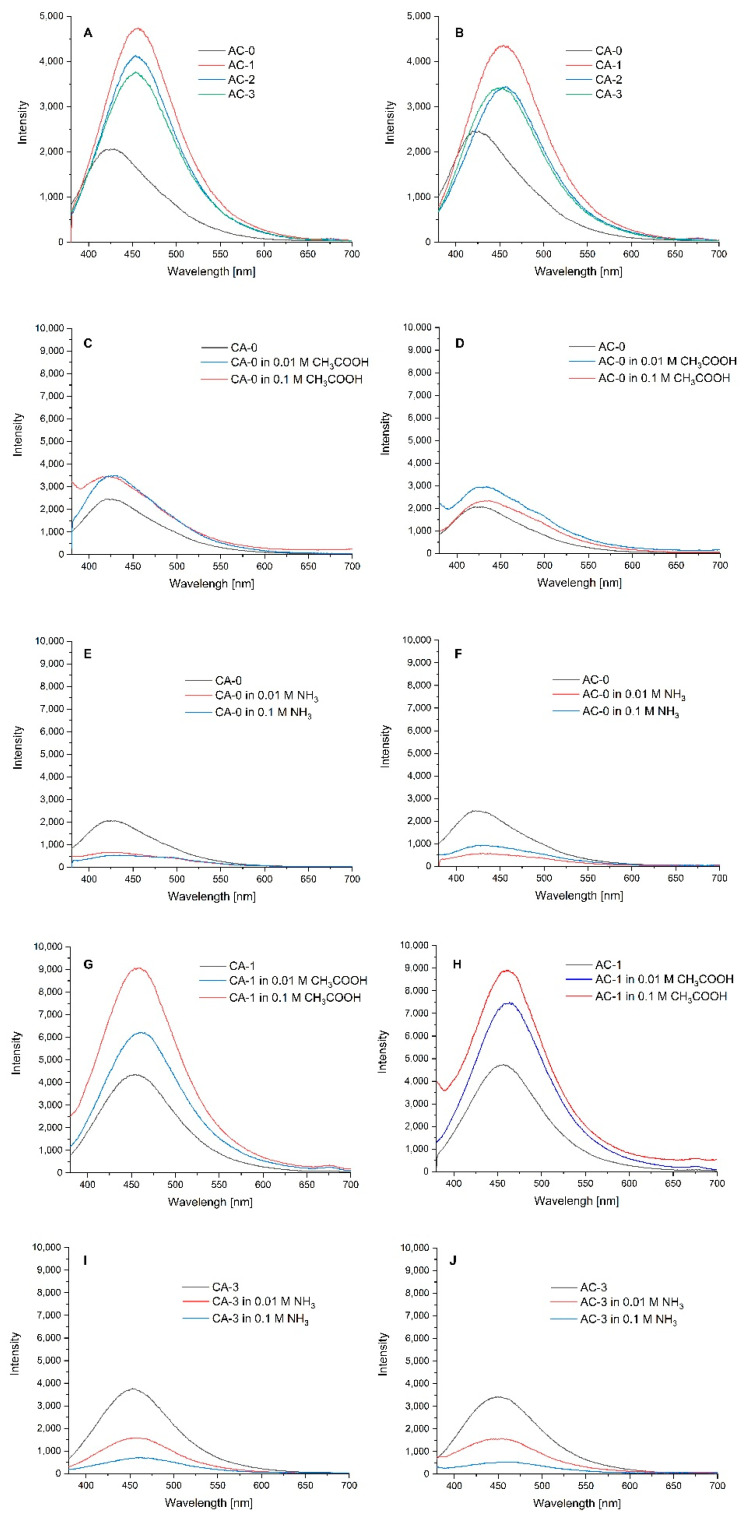
Emissions for CA- (**A**) and AC-series (**B**) composites; CA-0 (**C**), AC-0 (**D**), CA-1 (**G**) and AC-1 (**H**) composites when affected by acetic acid; CA-0 (**E**), AC-0 (**F**), CA-3 (**I**) and AC-3 (**J**) composites when affected by ammonia.

A comparison of the emission properties of the composites produced and the effects of an acid and base are shown in Figure 3A–J. For the CA-series composites, the presence of capsules increased the emission intensity and shifted the band. However, for both series (CA and AC), the highest intensity was observed for composites containing emulsion no. 1 (CA-1 and AC-1). This is due to the fact that anthocyans, responsible for emissions from the hibiscus extract, are more soluble in water. The plant extracts exhibit fluorescence, and this emission is dependent on pH [43]. For this reason, the composites were subjected to the presence of acetic acid and ammonia, and their emissions were measured. The results showed that the now-binary matrix produces an increased emission intensity when affected by an acid and decreased emissions when affected by a base. The composites functionalised with capsules produced increased emission intensities when affected by acids in general and by higher acid concentrations in particular. Tavassoli et al. [44] showed that anthocyanins in composite films changed colour in response to changes in pH. Changes in the emission intensity may occur, e.g., as a result of acid–base tautometry taking place in organic dyes under the influence of pH [45]. The emission tests performed suggest that the bionanocomposites produced are sensitive to pH changes and, therefore, can serve as food freshness sensors. 

### 2.4. Scanning Electron Microscopy (SEM) of the Obtained Composites

**Figure 4:**
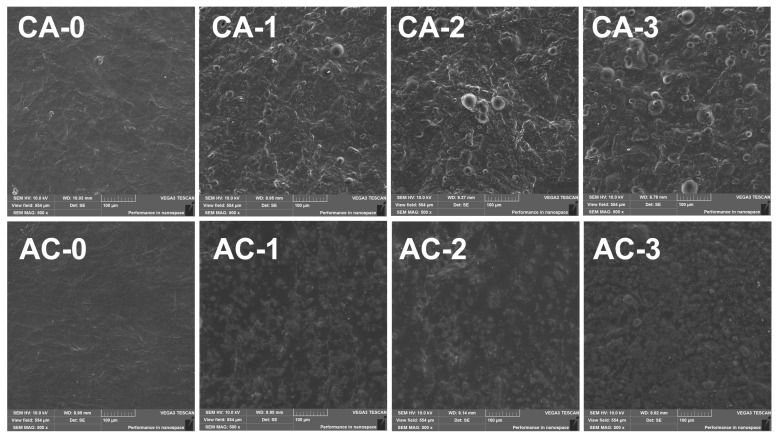
SEM images for the CA- and AC-series composites at 500× magnification.

The SEM images of the original and modified composites are shown in Figure 4. It can be noted in the visualisation that functionalisation did have an effect on the film structure. The surface of the unmodified films is smooth with visible fibrous structures, which may be the result of alginate and chitosan complexing, as can be seen in both the first and second composite variants in Figure 4. This phenomenon is consistent with the results from other researchers, who report that as chitosan content increases, so does the structural irregularity and porosity of the films [46]. After functionalisation, the presence of fully formed micro/nanocapsules with internal diameters between 100 and 1000 nm can be observed in the film structure. The micro/nanocapsules produced in the first series are numerous and have regular, spherical shapes. It can also be seen in the images that the spherical objects have a darkened area in the middle, suggesting that their structure remains intact and their contents have not migrated to the polysaccharide matrix space. Series two (Figure 4) is instead characterised by multiple aggregates, a branched structure and free, darkened areas in the film matrix. The microscopic images of the second series are much darker, which may result from the fact that a macroscopic oil leak on the film surface was observed, which may have disrupted the process of sample observation during their excitation with electrons. The results are consistent with the FTIR spectral analysis results, where a higher band intensity was observed at a wavelength of 1744 cm^−1^, which is caused by fatty acid carboxylic groups. It can therefore be concluded that the encapsulation conducted in series two led to less beneficial results than in the CA series. The micro/nanocapsules are not present in as great numbers as in series one. Furthermore, no destruction of the micro/nanocapsule structure caused by the electron beam was observed. 

### 2.5. Water Content and Water Solubility of Obtained Composites

The aqueous properties of the composites are summarised in Table 1. The water content (WC) in the control samples was 11.23% for CA-0 and 10.18% for AC-0, while the differences between them were statistically significant. Emulsion-modified films in both series were characterised by a lower water content. This phenomenon can be attributed to the presence of olive oil in the emulsion, as it has a hydrophobic character, which caused the films to contain less water. In series two, the water content in samples AC-1, AC-2 and AC-3 was 8.68%, 8.56% and 8.5%, respectively, while the differences between AC-2 and AC-3 were not statistically significant. The changes in water content in the composites may be caused by interactions between the sodium alginate and chitosan molecules, as well as interactions between olive oil and chitosan [47]. The water solubility (WS) for unmodified samples was 68.47% for CA-0 and 66.86% for AC-0; the differences were not statistically significant. Adding an emulsion to the film reduced the film’s water solubility by approx. 15–20% for both series. The water solubility for the polysaccharide materials produced is connected to several phenomena, i.e., olive oil and glycerine migration, as well as the water solubility of sodium alginate. All of the above components could migrate to water, leading to a loss in mass. This was observed visually and by analysing the microscopic images. 

### 2.6. Thermal Properties

The thermal properties, investigated by means of DSC analysis, are summarised in Table 2. The DSC observations identified an endothermic transformation at a temperature of approx. 100 °C, followed by an exothermic reaction at approx. 300 °C. The endothermic transition was caused by the absorption of heat and the resulting evaporation of water from the composites. In most cases, the test composites were characterised by two endothermic transitions, except for composite CA-1, which showed three decompositions. This indicates that it has the best thermal stability among the test composites. The control composites CA-0 and CA-1 were characterised by two transitions, with T_m_ during the first transition being 107.71 °C for CA-0 and 95.41 °C for AC-0. It can thus be suspected that the order in which the polysaccharides combine with one another affects the thermal stability of the resulting composite. When comparing the functionalised CA series with AC, it can be noted that the AC series exhibited higher T_m_ values for the first decomposition than the CA series. The DSC results also suggest that the filler replaces water in the hydrogel matrix and improves its thermal stability, which is beneficial for the delivery of encapsulated bioactive compounds [48]. Based on the DSC results, it can be concluded that the presence of nano/microcapsules improves the composite’s stability. The results are in agreement with previous data, as encapsulation leads to improved thermal stability, regardless of the used compounds.

The thermal decomposition process of the composites was assessed by thermogravimetric analysis, with the results shown in Table 3. The analysis showed that the control composites (CA-0 and AC-0) were characterised by two transitions. For CA-0, they occurred Within the 46.37–169.94 °C temperature range during the first transition and 171.73–495.19 °C during the second. For the AC-0 composite, the first decomposition stage occurred within the 41.73–171.10 °C temperature range during the first transition and 172.46–499.83 °C during the second. The composites enriched with emulsions showed up to four decomposition stages, except composite CA-3, for which three decomposition stages were recorded. Based on the results, it can be surmised that the composites containing the ozonated olive oil emulsion showed lower thermal stability. In series two, composites AC 1–3 showed T_m_ values higher than those of the control, which may be the result of the stronger immobilisation of water particles between the chitosan chains. Additionally, during the combining of the polymers, a reaction occurs between the amine groups of chitosan and the carboxylic groups of sodium alginate. Interactions between polar groups and these functional groups of polysaccharides are impeded, and fewer groups in the polysaccharides can react with water molecules, which may lead to a lower water absorption capacity [49]. According to literature data, thermal degradation of chitosan films occurs in two stages that involve multiple phenomena, i.e., solvent evaporation (acetic acid and water) and processes including saccharide ring dehydration, depolymerisation and decomposition of the acetylated and deacetylated polymer units [50].

### 2.7. Mechanical Properties of the Films

The thickness of the obtained films and their mechanical properties are shown in Table 4. The control films (CA-0 and AC-0) were about 31–59% less thick than the other analysed films. The increased thickness of films with nanocapsules implemented (CA-1, CA-2, CA-3 and AC-1, AC-2, AC-3) can be attributed to the enrichment of the solid content in the samples [51].

Tensile strength (TS) is the force required to break a film of specified dimensions. The higher the tensile strength, the wider the applicability of the tested composites to packaging designed to protect the product from mechanical damage. The addition of nanoparticles to the polysaccharide matrix worsened its mechanical properties. In all cases, a decrease in the tensile strength and elongation of the films were observed. Although the addition of nanoparticles weakened the polysaccharide structure, the TS values were higher than those of low-density polyethylene (LDPE) (16.5 MPa). The elongation of some of the obtained nanocomposites (CA-1, CA-2 and CA-3) was higher than that of polyester (PE) (18%) and polyvinylidene chloride (PVDC) (19%) [52]. 

The changes in the mechanical properties of the functionalised films result from the hydrophobicity of olive oil, which contains hibiscus flower extract and significantly restricts interactions with the hydrophilic phase of the polymer hydrogel. This leads to poor dispersion and, ultimately, to the separation of the phases, both in the solution and in the final dried film [53]. The incompatibility leads to weaker mechanical properties of the resulting films. The method of encapsulating the hibiscus extract had no statistically significant effect on the stretchability of the dried polymer matrix and only a minor effect on its strength. It is, however, interesting that the matrix functionalisation process was dependent on the order in which the polymers were added to the hydrogel. In the first series, sodium alginate was added to chitosan gel, while in the second series, the order was reversed. While the mechanical properties of the binary polymer matrix did not change as a result of changing the order in which the polymers were combined, the nano/microcapsule addition to this matrix did affect its properties. A much more pronounced effect of functionalisation was noted for the alginate–chitosan matrix. This behaviour can be explained by the weakening of the film structure due to oil leakage on the film surface, which was confirmed by microscopic observations (Figure 4).

There are also reports in the literature that indicate that the lowering of all the tensile parameters (strength, elongation at rupture) of chitosan films is the expected effect after incorporating a liquid lipid into the system [47].

### 2.8. Optical Properties of Composites

The optical properties, including the colour and opacity, of the composites are summarised in Table 5. The control films are characterised by high brightness; for CA-0, the L* value was 97.28, while for AC-0, it was 97.62; the differences between these samples were statistically significant. Introducing the olive oil emulsion with added hibiscus flower extract to the composites led to changes in all colour parameters. The a* parameter showed negative values only in the control samples CA-0 and AC-0, for which it was −0.54 and −0.48, respectively. The negative value of the chromaticity coordinate a* indicates that the film surface showed a colour in the direction of shades of green. Similar results for chitosan- and sodium alginate-based films were obtained by Khachatryan et al. [39], who developed nanocomposites based on chitosan and sodium alginate and demonstrated that the a* values for chitosan/alginate films were characterised by a marked dominance of the colour green [39]. Similar observations were noted for the b* parameter, with the lowest values of 8.09 ± 0.10 and 7.11 ± 0.01 shown by control samples, and the differences between them were statistically significant. On this basis, it can be surmised that the differences resulted from the order in which the polysaccharides were added. The colour parameter changes can be attributed to the olive oil and the presence of the encapsulated hibiscus flower extract. Furthermore, the effect on changes in the a* and b* parameters of the control films for both groups can also be attributed to the natural colour of the polysaccharides. The composite opacity was determined for absorbance at a wavelength of 600 nm (Table 5). The opacity results indicate that the control film of series one is characterised by high transparency and clarity, and its value was 3.85, while in the reverse series, the opacity was 5.74. This difference may stem from the order in which the polysaccharides were added, while the complex in which chitosan was added to alginate may have formed more solid fractions, which led to higher turbidity in the sample. The higher O value for the composites containing olive oil-based emulsions with hibiscus flower extracts indicates a lower transparency level in the films. For the films with emulsions, the opacity results were 8.78 and 9.18, respectively, while for the sample with ozonated olive oil, it was 7.95. On the other hand, the results for series two were 8.81, 8.29 and 7.74, respectively. However, for the films with ozonated olive oil, the opacity was lower in both series. These values indicate that even a minor addition of an emulsion containing an encapsulated plant extract can prevent UV light penetration, compared to an unmodified binary polysaccharide matrix. The opacity level in food packaging materials is important for food products that require protection from light. This applies to food products that can degrade in light-induced reactions [54].

**Figure 5:**
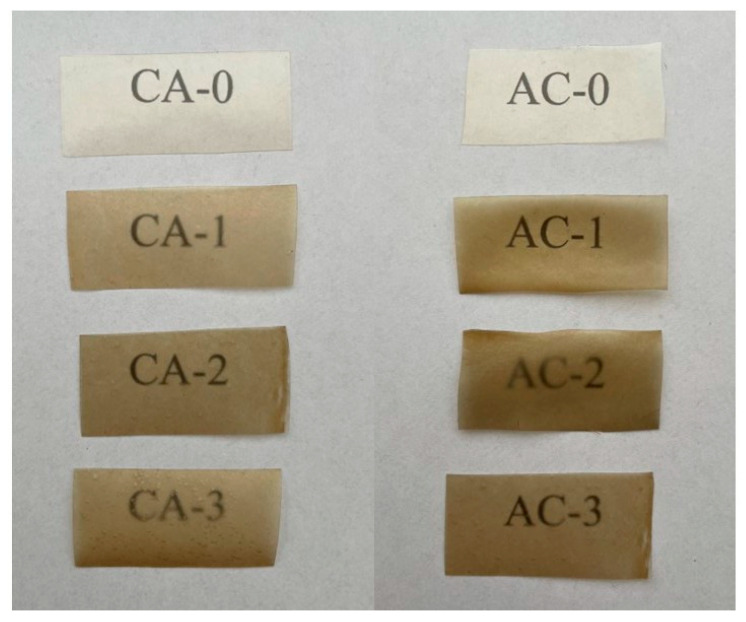
Appearance of the obtained composites.

The emulsions produced were characterised by a pink to red colour. Dried hibiscus leaves contain large amounts of anthocyans, which confer an intense purple or red colour to its aqueous or ethanol extracts [55]. The appearance of the films produced is shown in Figure 5. Based on the visual assessment, it can be concluded that the chitosan-alginate and alginate-chitosan composites were colourless and showed slight turbidity, which can be attributed to the interactions between the protonated amine groups of chitosan and carboxylic groups of alginate, which lead to the formation of aggregates and insoluble heterogeneous floccules during complexing [56]. For the modified composites containing micro/nanocapsules, the films were coloured in shades of grey (Figure 5). Enclosing the hibiscus flower extract in the emulsion and subsequently in the polysaccharide matrix definitely had an effect on the colour of the extract of the emulsions produced. 

## 3. Materials and Methods

### 3.1. Materials

The following chemical reagents were used to produce the nanocomposites: chitosan (high molecular weight: 310,000–375,000 Da; degree of deacetylation > 75%) from shrimp shells (Sigma-Aldrich, Poznań, Poland), sodium alginate (Sigma-Aldrich, Poznań, Poland), acetic acid (99,5%, Chempur, Piekary Śląskie, Poland), glycerin (99.5%, Chempur, Piekary Śląskie, Poland), deionised water, extra virgin olive oil, ozonated extra virgin olive oil (with an ozone content of 1.11 ± 0.02 g in 100 g of oil, Scandia Cosmetics, Niepolomice, Poland) and dried hibiscus flower calyx (Agnex, Białystok, Poland). 

### 3.2. Methods

#### 3.2.1. Extraction and Emulsification

**Figure 6:**
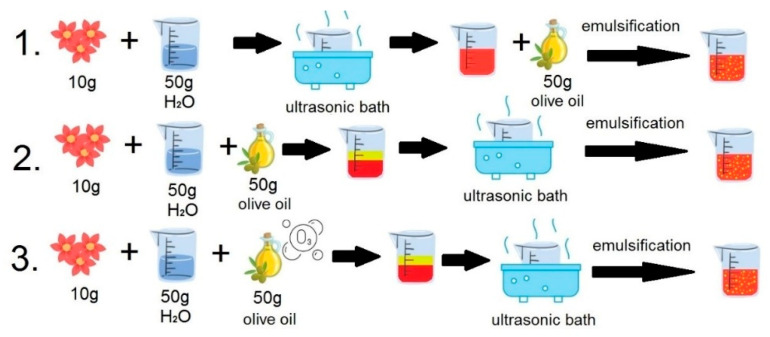
Diagram of the extraction and emulsification process.

Figure 6 shows a schematic of the extraction and emulsification process.

Emulsion 1: First, 50 g of deionised water was added to 10 g of dried hibiscus flowers, and the mixture was placed in an ultrasound bath (Sonic 10, Polsonic, Warsaw, Poland) for 10 min at 40 °C. After filtration, 50 g of oil was added to the filtrate, and emulsification was performed using a 20 kHz low-frequency ultrasonic processor (Sonopuls HD 4200, Bandelin, Berlin, Germany) for 5 min.

Emulsion 2: First, 50 g of deionised water and 50 g of oil were added to 10 g of dried hibiscus flowers, and the mixture was placed in an ultrasound bath (Sonic 10, Polsonic, Warsaw, Poland) for 10 min at room temperature (25 ± 2 °C). After the hibiscus flowers were removed, the water and oil mixture was emulsified using a 20 kHz low-frequency ultrasonic processor (Sonopuls HD 4200, Bandelin, Berlin, Germany) for 5 min.

Emulsion 3: First, 50 g of deionised water and 50 g of ozonated oil were added to 10 g of dried hibiscus flowers, and the mixture was placed in an ultrasound bath (Sonic 10, Polsonic, Warsaw, Poland) for 10 min at 40 °C. After the hibiscus flowers were removed, the water and oil mixture was emulsified using a 20 kHz low-frequency ultrasonic processor (Sonopuls HD 4200, Bandelin, Berlin, Germany) for 5 min. 

#### 3.2.2. Preparation of Polysaccharide Gels

A suspension of 40.0 g of sodium alginate in 940 g of water was prepared. The resulting suspension was stirred with a magnetic stirrer (Heidolph RZR 2020, Heidolph Instruments GmbH & Co. KG, Schwabach, Deutschland) at 70 °C, 700 RPM, until a homogeneous gel was obtained. Then, 20 g of glycerine was added as a plasticiser. A 2% sodium alginate hydrogel was thus obtained.

Similarly, 2000 g of 2% chitosan hydrogel was prepared by weighing out 40.0 g chitosan and dissolving it in 940 g of 0.5% acetic acid solution. The mixture was placed on a magnetic stirrer (700 rpm, temperature: 70 °C; Heidolph RZR 2020, Heidolph Instruments GmbH & Co. KG, Schwabach, Deutschland) until a clear gel was obtained.

#### 3.2.3. Preparation of Films

**Figure 7:**
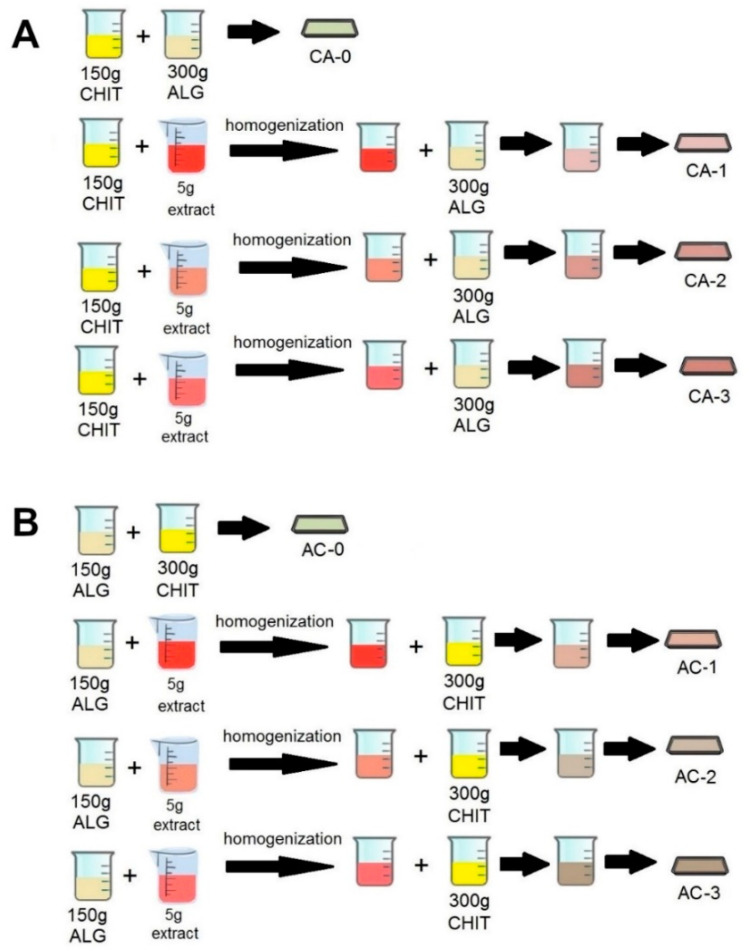
Diagram of the CA- (**A**) and AC-series (**B**) film preparation process.

Figure 7 shows a schematic of the process of preparing CA- and AC-series films.

##### CA Series

First, 150 g of 2% chitosan hydrogel was added to a 300 g 2% suspension of sodium alginate while mixing with a mechanical homogeniser (Polytron PT 2500 E, Kinematica AG, Malters, Switzerland) for 15 min. The resulting hydrogel was poured into a 250 × 250 mm polypropylene tray and dried at 25 °C (±2 °C) for 48 h. The resulting flexible film was designated CA-0.

Next, 5.0 g of emulsion 1 was added to a 150 g 2% chitosan hydrogel and mixed with a mechanical homogeniser (Polytron PT 2500 E, Kinematica AG, Malters, Switzerland) until a uniform mixture was produced, and the resulting hydrogel was added to a 300 g 2% sodium alginate suspension while stirring with the mechanical homogeniser for a further 15 min. The resulting hydrogel was poured into a 250 × 200 mm polypropylene tray and dried at room temperature (25 ± 2 °C) for 48 h. The resulting flexible film was designated CA-1.

Samples designated CA-2 and CA-3 were prepared in a similar manner using emulsions 2 and 3, respectively.

##### AC Series

A 150 g 2% sodium alginate hydrogel was added to a 300 g 2% suspension of chitosan while mixing with a mechanical homogeniser (Polytron PT 2500 E, Kinematica AG, Malters, Switzerland) for a further 15 min. The resulting hydrogel was poured into a 250 mm × 200 mm polypropylene tray and dried at room temperature (25 ± 2 °C) for 48 h. The resulting flexible film was designated AC-0.

Next, 5.0 g of emulsion 1 was added to 150 g of the 2% sodium alginate hydrogel and mixed with a mechanical homogeniser (Polytron PT 2500 E, Kinematica AG, Malters, Switzerland) until a uniform mixture was produced, and the resulting hydrogel was added to a 300 g 2% chitosan suspension while stirring with the mechanical homogeniser for a further 15 min. The resulting hydrogel was poured into a 250 mm × 200 mm polypropylene tray and dried at 25 °C (±2 °C) for 48 h. The resulting flexible film was designated AC-1. 

Samples designated AC-2 and AC-3 were prepared in a similar manner using emulsions 2 and 3, respectively.

#### 3.2.4. Water Content and Solubility

Three 20 mm × 20 mm squares were cut out at random from each of the films at a distance of at least 10 mm from the film edge. The squares were weighed (to 0.0001 g), and their initial weights (M_1_) were recorded. The samples were then dried in an oven at 130 °C for 2 h to determine the starting dry weights (M_2_). After drying, the films were immersed in 30 mL of distilled water, covered tightly and stored for 24 h at room temperature (25 °C ± 2 °C). After this time, all samples were dried on filter paper and subsequently dried in an oven at a temperature of 130 °C for 2 h to determine the end non-dissolved dry matter weights (M_3_). For each film sample, three measurements were performed to calculate the mean value. Water content and film solubility in water were calculated using the following formulae:

Water content (WC) = M_1_ − M_2_/M_1_ × 100% (1)

Water solubility (WS) =M_2_ − M_3_/M_2_ × 100% (2)

#### 3.2.5. Mechanical Properties of Composites

The analysis was performed in accordance with ISO Standards [57]. The films were cut into 35 mm × 6 mm strips and placed in grips. The initial distance between the grips was 20 mm, and the peel rate was 2 mm/min. Tensile strength (TS) was calculated by dividing the maximum force at break of the film by the cross-sectional area of the film. The percent elongation at break (EAB) was calculated by dividing the elongation at break point by the initial measurement length and multiplying by 100 [51,58]. The results reported are averages of ten repetitions.

#### 3.2.6. Thickness Measurement

The thickness of composites was measured with a micrometer, catalogue no.: 805.1301 (Sylvac SA, Crissier, Switzerland), with a 0.001 mm resolution. The sample thickness was the average of five measurements performed in various places within the gauge length area [59].

#### 3.2.7. Surface Colour Measurements

The surface colour was measured with the use of Konica MINOLTA CM-3500d equipment (Konica Minolta Inc., Tokyo, Japan) using a reference D65 illuminant/10ᵒ observer with a 3 mm diameter window. The results were expressed using the CIELab system. The following parameters were determined: L* (L* = 0 black, L* = 100 white); a*—share of green (a* < 0) or red colour (a* > 0); b*—share of blue (b* < 0) or yellow (b* > 0) [51]. The measurements were taken on a white background standard. The experiment was repeated 5 times.

#### 3.2.8. FTIR-ATR Spectrophotometry

The FTIR-ATR spectra of the obtained composites were analysed using a MATTSON 3000 spectrophotometer (Madison, WI, USA) n the 4000–700 cm^−1^ range at a resolution of 4 cm^−1^. The spectrophotometer was equipped with a 30SPEC 30 Degree Reflectance adapter (MIRacle ATR, PIKE Technologies Inc., Madison, WI, USA).

#### 3.2.9. UV-Vis Absorption Spectrophotometry and Opacity

The UV-Vis absorption spectra of the composites were recorded using a Shimadzu 2101 (Shimadzu, Kyoto, Japan) scanning spectrophotometer in the 200–800 nm range. To carry out the measurement, pieces of the films were placed in a 10 mL quartz cuvette, with an empty cuvette used as a reference. The opacity (O) was also determined at a wavelength of 600 nm according to the following equation:

$$\mathrm{Opacity}=\frac{\mathrm{Abs}600}{x}$$

where Abs 600 is the absorbance value at 600 nm, and x is the film thickness (mm).

#### 3.2.10. Photoluminescence Spectroscopy

**Figure 8:**
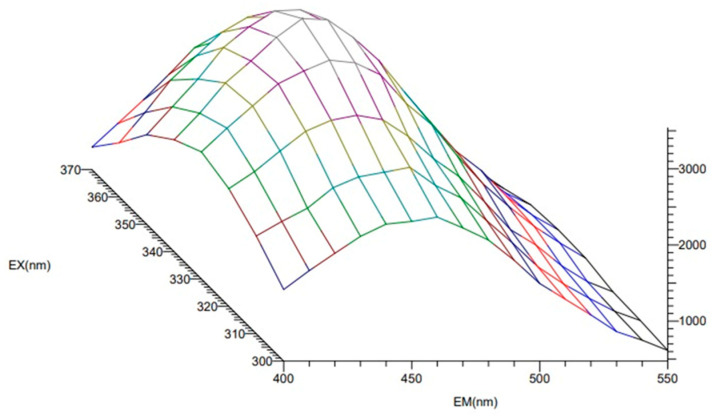
The 3D spectrum for the CA-1 sample.

Photoluminescence measurements for the films were carried out at room temperature using a HITACHI F7000 spectrophotometer (Hitachi Co. Ltd., Tokyo, Japan). The emission spectra of the samples were measured at an excitation wavelength of 360 nm. The wavelength was chosen based on the 3D spectrum (emission intensity measurement depending on excitation wavelength), which indicates that the samples exhibit the highest emission intensity at an excitation wavelength of 360 nm. Figure 8 shows a sample 3D spectrum for the CA-1 film.

The effects of acetic acid and ammonia on the emission properties were measured for films placed in the respective aqueous solutions for 5 s. The films were then dried, and their emissions were measured.

#### 3.2.11. Differential Scanning Calorimetry (DSC)

The thermal transformations of the composites before and after modifications were examined using a STARe System DSC 3 (Mettler Toledo, Greifensee, Switzerland) differential scanning colorimeter. About 4 mg of each sample was weighed and enclosed in an aluminium crucible with a cover. Then, the samples were heated from 30 °C to 200 °C at a rate of 10 °C/min. The inert gas flow was 100 mL/min. An empty aluminium crucible with a cover was used as a reference. The registered DSC curves were analysed using Mettler Toledo STARe Evaluation Software.

#### 3.2.12. Thermogravimetric Analysis (TGA)

Thermal stability analysis for the materials was performed on a STARe System TGA/DSC 3+ (Mettler Toledo, Greifensee, Switzerland) thermal analyser using two methods: thermogravimetric (TG) and differential thermogravimetric (DTG). Composite samples in open aluminium oxide crucibles were placed in a measurement chamber and heated from 30 to 500 °C at a constant heating rate of 10 °C/min. The measurements were performed in an inert gas environment at a flow rate of 50 mL/min. The registered thermograms were analysed using Mettler Toledo STARe Evaluation Software. The measurements were performed in two replications.

#### 3.2.13. Scanning Electron Microscopy (SEM)

The surface morphology of the polysaccharide composites was characterised using a Tescan Vega 3 SBU (Tescan, Brno, Czech Republic) scanning electron microscope. Scanning electron microscopy (SEM) was used to check morphological changes in the control polysaccharide composites and emulsion-functionalised composites. Film samples were fixed on tables using carbon tape, without covering them with a conducting material. Microscopic observations were performed under high vacuum and at an accelerating voltage of 5 kV.

#### 3.2.14. Statistical Analysis

The experimental data were subjected to an analysis of variance at the significance level 0.05 using Statistica v. 8.0 software (Statsoft, Inc., Tulsa, OK, USA). The Fisher test was used for the determination of statistically significant differences.

## 4. Conclusions

Binary films based on two polysaccharides—chitosan and alginate—were successfully produced and functionalised with an olive oil emulsion or ozonated olive oil with a hibiscus flower extract. The addition of olive oil and the hibiscus flower emulsion affected the parameters of the resulting films, but the functionalisation did not lead to chemical interactions with polymers and therefore did not disrupt their structures, which was demonstrated by the FTIR spectroscopy results. SEM imaging confirmed that the desired micro/nanostructures, which were not observed in the control films, were obtained. A comparison of the two series demonstrated that the CA series was characterised by a superior encapsulation capability. The produced materials were sensitive to acid–base changes. The results suggest that the bionanocomposites can be used as active and/or smart materials. These can potentially find use as food product freshness sensors, although it is necessary to verify their effectiveness on model food products as a part of further research.

## Figures and Tables

**Table 1:** Water content and solubility of composites.

Sample	Water Content	Water Solubility
CA-0	11.23 ± 0.18 ^a^	68.47 ± 4.36 ^a^
CA-1	9.72 ± 0.09 ^b^	47.54 ± 0.98 ^b^
CA-2	8.57 ± 0.41 ^c^	48.33 ± 0.40 ^b^
CA-3	8.55 ± 0.13 ^c^	53.09 ± 1.65 ^c^
AC-0	10.18 ± 0.26 ^d^	66.86 ± 3.78 ^a^
AC-1	8.68 ± 0.06 ^c^	50.03 ± 0.80 ^bc^
AC-2	8.56 ± 0.16 ^cd^	49.97 ± 2.24 ^bc^
AC-3	8.50 ± 0.17 ^c^	47.55 ± 0.73 ^b^

The same superscript letters in each column demonstrate a lack of significant difference between values (*p* < 0.05). Values are expressed as mean ± SD.

**Table 2:** Comparison of DSC thermal properties.

	1st Decomposition		2nd Decomposition		3rd Decomposition	
Film Type	T_m_ (°C)	ΔT (°C)	T_m_ (°C)	ΔT (°C)	T_m_ (°C)	ΔT (°C)
CA-0	107.71	59.73–86.83	-	-	-	-
CA-1	97.39	96.84–98.78	127.04	126.55–28.57	191.54	191.05–192.65
CA-2	103.23	73.24–20.89	-	-	-	-
CA-3	108.38	59.77–59.59	184.39	167.69–199.36	-	-
AC-0	95.41	52.36–133.84	173.59	167.26–97.77	-	-
AC-1	111.69	104.23–72.4	-	-	-	-
AC-2	109.69	74.03–157.6	-	-	-	-
AC-3	115.69	77.04–147.64	186.35	167.47–199.77	-	-

T_m_—peak temperature; ΔT—temperature limits.

**Table 3:** Comparison of thermal properties (Tm—maximum temperature of decomposition; ΔT—temperature range of decomposition).

	1st Decomposition		2nd Decomposition		3rd Decomposition		4th Decomposition	
Film Type	T_m_ (°C)	ΔT (°C)	T_m_ (°C)	ΔT (°C)	T_m_ (°C)	ΔT (°C)	T_m_ (°C)	ΔT (°C)
CA-0	114.27	46.37–169.94	222.92	171.73–495.19	-	-	-	-
CA-1	108.21	34.56–175.23	211.57	177.40–309.23	331.23	310.05–387.19	463.18–477.76	388.50–499.84
CA-2	109.16	42.77–178.12	216.56	180.83–311.89	334.09	312.08–393.20	472.88	396.07–499.87
CA-3	109.67	40.23–177.35	212.65	178.98–388.40	468.70	389.71–499.80	-	-
AC-0	109.82	41.73–171.10	216.39	172.46–499.83	-	-	-	-
AC-1	117.55	37.60–174.71	215.59–238.42	175.79–312.17	341.19	312.17–385.69	471.70	385.69–499.94
AC-2	119.72	38.72–174.34	215.93–238.02	175.16–315.76	344.10	316.02–386.36	473.89	386.62–499.84
AC-3	118.27	33.02–178.34	215.98–240.76	179.42–317.15	335.45	317.68–394.76	475.75	395.01–499.85

T_m_—peak temperature; ΔT—temperature limits.

**Table 4:** Properties and thickness of the films.

Sample	Thickness (mm)	TS (MPa)	EAB (%)
CA-0	0.144 ± 0.007 ^c^	61.79 ± 6.74 ^a^	31.28 ± 4.91 ^a^
CA-1	0.192 ± 0.005 ^b^	43.22 ± 3.12 ^b^	22.61 ± 1.94 ^b^
CA-2	0.202 ± 0.006 ^a^	33.80 ± 5.91 ^c^	23.93 ± 3.39 ^b^
CA-3	0.189 ± 0.010 ^b^	36.40 ± 7.87 ^c^	20.13 ± 4.96 ^b^
AC-0	0.145 ± 0.008 ^b^	61.30 ± 4.34 ^a^	31.82 ± 3.22 ^a^
AC-1	0.217 ± 0.015 ^a^	32.29 ± 4.56 ^b^	15.06 ± 5.18 ^b^
AC-2	0.220 ± 0.016 ^a^	29.31 ± 5.23 ^b^	13.75 ± 1.63 ^b^
AC-3	0.231 ± 0.018 ^a^	28.40 ± 4.62 ^b^	16.04 ± 3.05 ^b^

Different superscript letters in column denote mean values that statistically differ from one another (Fisher’s test, at α = 0.05). TS—tensile strength; EAB—percent elongation at break.

**Table 5:** Colour parameters and opacity of films.

Sample	L* (D65)	a* (D65)	b* (D65)	Opacity
CA-0	97.28 ± 0.09 ^b^	−0.54 ± 0.02 ^g^	8.09 ± 0.10 ^f^	3.85
CA-1	81.64 ± 0.21 ^d^	0.82 ± 0.05 ^c^	17.22 ± 0.12 ^b^	8.78
CA-2	75.48 ± 0.11 ^e^	0.24 ± 0.07 ^d^	15.97 ± 0.15 ^c^	9.18
CA-3	83.10 ± 0.15 ^c^	0.84 ± 0.01 ^c^	16.05 ± 0.11 ^c^	7.95
AC-0	97.62 ± 0.10 ^a^	−0.48 ± 0.01 ^f^	7.11 ± 0.01 ^g^	5.74
AC-1	75.09 ± 0.31 ^f^	0.97 ± 0.00 ^b^	15.75 ± 0.06 ^d^	8.81
AC-2	69.43 ± 0.14 ^g^	0.17 ± 0.03 ^e^	12.62 ± 0.14 ^e^	8.29
AC-3	75.13 ± 0.31 ^f^	2.48 ± 0.04 ^a^	20.00 ± 0.08 ^a^	7.74

The same superscript letters in each column demonstrate a lack of significant difference between values (*p* < 0.05). Values are expressed as mean ± SD.

## Data Availability

The data presented in this study are available upon request from the corresponding author.
